# InterMitoBase: An annotated database and analysis platform of protein-protein interactions for human mitochondria

**DOI:** 10.1186/1471-2164-12-335

**Published:** 2011-06-30

**Authors:** Zuguang Gu, Jie Li, Song Gao, Ming Gong, Junling Wang, Hua Xu, Chenyu Zhang, Jin Wang

**Affiliations:** 1 The State Key Laboratory of Pharmaceutical Biotechnology and Jiangsu Engineering Research Center for MicroRNA Biology and Biotechnology, School of Life Science, Nanjing University, Nanjing 210093, China

## Abstract

**Background:**

The mitochondrion is an essential organelle which plays important roles in diverse biological processes, such as metabolism, apoptosis, signal transduction and cell cycle. Characterizing protein-protein interactions (PPIs) that execute mitochondrial functions is fundamental in understanding the mechanisms underlying biological functions and diseases associated with mitochondria. Investigations examining mitochondria are expanding to the system level because of the accumulation of mitochondrial proteomes and human interactome. Consequently, the development of a database that provides the entire protein interaction map of the human mitochondrion is urgently required.

**Results:**

InterMitoBase provides a comprehensive interactome of human mitochondria. It contains the PPIs in biological pathways mediated by mitochondrial proteins, the PPIs between mitochondrial proteins and non-mitochondrial proteins as well as the PPIs between mitochondrial proteins. The current version of InterMitoBase covers 5,883 non-redundant PPIs of 2,813 proteins integrated from a wide range of resources including PubMed, KEGG, BioGRID, HPRD, DIP and IntAct. Comprehensive curations have been made on the interactions derived from PubMed. All the interactions in InterMitoBase are annotated according to the information collected from their original sources, GenBank and GO. Additionally, InterMitoBase features a user-friendly graphic visualization platform to present functional and topological analysis of PPI networks identified. This should aid researchers in the study of underlying biological properties.

**Conclusions:**

InterMitoBase is designed as an integrated PPI database which provides the most up-to-date PPI information for human mitochondria. It also works as a platform by integrating several on-line tools for the PPI analysis. As an analysis platform and as a PPI database, InterMitoBase will be an important database for the study of mitochondria biochemistry, and should be particularly helpful in comprehensive analyses of complex biological mechanisms underlying mitochondrial functions.

## Background

The mitochondrion is an essential organelle in eukaryotic cells that plays important roles in a variety of important processes such as apoptosis, signal transduction and cell cycle [1]. Mitochondrial dysfunction is linked to many common diseases including heart disease, diabetes, Parkinson disease and dementia. To understand the mechanism underlying the biological functions and diseases associated with the mitochondria, it is important to determine protein-protein interactions (PPIs) that facilitate mitochondrial functions.

The extensive use of experimental approaches including 2D gel electrophoresis and mass spectrometry, has led to the construction of many databases for mitochondrial proteomics, such as MitoCarta [2], MitoProteome [3], MitoP2 [4] and HMPDb [5]. Increasing interest in mitochondrial proteomics is promoting studies on PPIs of mitochondria at a systems level. By unraveling the interplays between mitochondrial proteins and mitochondrial/non-mitochondrial proteins, the entire interaction map that contributes to mitochondrial functions will be revealed.

Although several PPI databases have been distributed, such as HPRD [6], BioGRID [7], IntAct [8] and DIP [9], there are very few PPI databases that are designed specifically for mitochondria. MitoInteractome [10] is a representative interaction database for mitochondria. However, this database only contains interactions between mitochondrial proteins which are predicted based on structural and homologous information. None of the interactions between mitochondrial proteins and non-mitochondrial proteins have been included. These types of interactions are very important for characterizing the mechanisms of mitochondrial function because they contain information about how the mitochondrion communicates with the intracellular environment. Therefore, it is necessary to construct a database covering the entire PPI map that characterizes the global mitochondrial functions.

Here, we have developed a database termed InterMitoBase, which covers the biological pathways mediated by mitochondrial proteins and the PPIs between mitochondrial and mitochondrial/non-mitochondrial proteins. The interactions in InterMitoBase are integrated from a wide range of resources including PubMed, KEGG [11], HPRD, BioGRID, IntAct and DIP, all of which are well annotated according to the information collected from their original sources GenBank and GO. InterMitoBase features as a user-friendly graphic visualization tool and provides functional and topological analysis of PPI networks that should facilitate an understanding of the underlying biological properties. As an analysis platform and a PPI database for human mitochondria, InterMitoBase should significantly aid researchers aiming to develop a comprehensive and deep understanding of complex mitochondrial functions.

## Construction and Content

InterMitoBase is designed as a web-based database providing graphic visualization of annotated PPI interactions that relate to human mitochondrial functions. It integrates the data from diverse sources such as MitoCarta, HPRD, the KEGG pathway database, PubMed and Gene Ontology [12]. Several on-line tools are also embedded in InterMitobase for functional and topological analyses of protein-protein networks.

### Protein-protein Interaction Data

**Figure 1:**
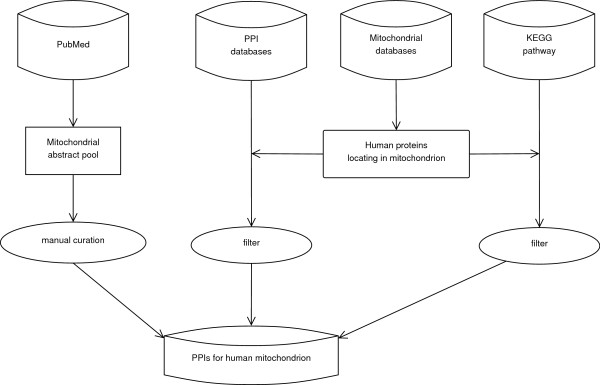
**Workflow of data collection**. The PPIs in InterMitoBase are collected from PubMed, KEGG, and other PPI databases of HPRD, BioGRID, IntAct and DIP.

The general process of data collection is illustrated in Figure 1. The interactions hidden in the literature were manually curated from 58,107 literature abstracts that were retrieved from PubMed (till July 19^th^, 2010) by querying "(mitochondrial [All Fields] OR mitochondria [All Fields] OR mitochondrion [All Fields]) AND humans [MeSH Terms] AND English [Language]". The interactions embedded from KEGG were then derived by collecting the biological pathways that contain at least one mitochondrial protein. Finally, the interactions between mitochondrial proteins and mitochondrial/non-mitochondrial proteins in the four PPI databases of BioGRID, HPRD, DIP and IntAct were integrated.

**Figure 2:**
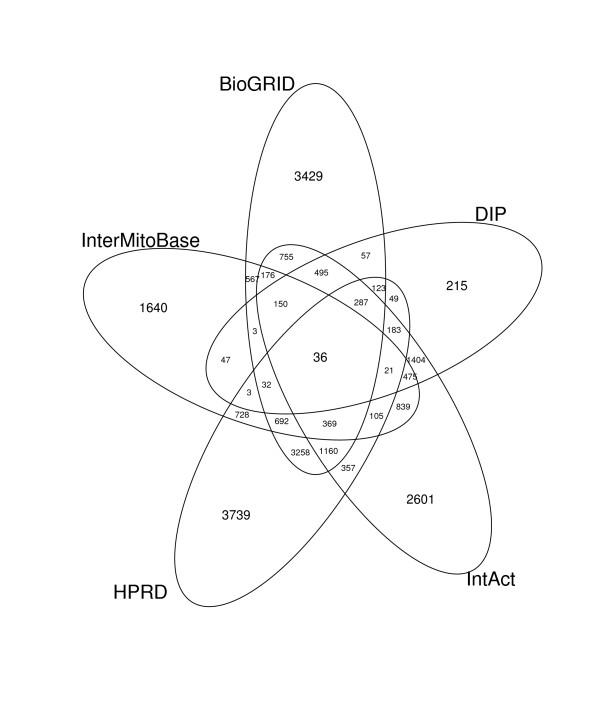
**Venn diagram of PPIs among InterMitoBase, BioGRID, DIP, HPRD and IntAct**.

The current version of InterMitoBase contains 5,883 PPIs of 2,813 proteins (490/2323 mitochondrial/non-mitochondrial proteins) collected from PubMed, KEGG, HPRD, BioGRID, IntAct and DIP. A comparison between the PPI databases of HPRD, BioGRID, IntAct and DIP, InterMitoBase is specifically designed for human mitochondria, which aims at all the possible PPIs that are closely related to human mitochondrial functions. This new database covers: (i) PPIs involved in biological pathways mediated by mitochondrial proteins; (ii) the PPIs between mitochondrial and non-mitochondrial proteins; and (iii) PPIs between mitochondrial and mitochondrial proteins. 1,640 PPIs in InterMitoBase are new ones that have not been covered by any of the four PPI databases (see Figure 2). More than 80% of the new interactions (1420/1640) have been manually curated from literature abstracts isolated from PubMed.

### Annotation of Protein-protein Interactions

PPIs in InterMitoBase are annotated as follows: 1) the basic information (e.g., name, transcript, genomic location and GO term) of the interacting proteins are annotated referring to GenBank and GO; 2) the information about subcellular location of every protein is provided according to MitoCarta, MitoProteome, MitoP2, HMPDb and UniProt; 3) the information about the direction and regulation type of each interaction is provided; 4) the full context that contains the interaction (i.e., the map of the KEGG pathway or the PubMed literature abstract) is supplied.

### Network Visualization and analysis

InterMitoBase provides an intuitionistic visualization of a protein-protein network formed by a set of PPIs. It also integrates a tool to analyze the degree distribution of the network. These will be helpful to uncover the global structural features and key elements of the network.

### Functional Enrichment Analysis

InterMitobase also embeds a tool that can evaluate the enriched functions of the proteins involved in a specific protein-protein network based on the Gene Ontology (GO) terms. The significance of the enriched functions is represented by the p-value that is evaluated following the Fisher's exact test. The false discovery rate (FDR) is controlled by the Benjamini-Hochberg process [13]. The analysis of the functional enrichment provides the general feature of the network function.

## System Architecture and Implementation

**Figure 3:**
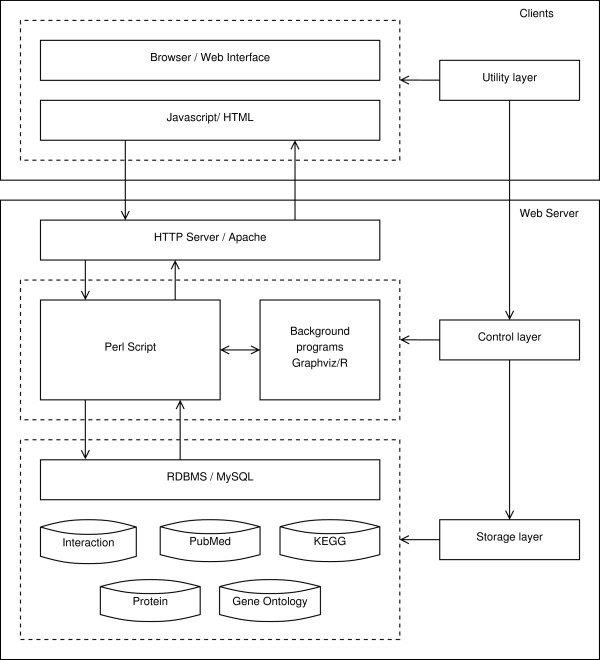
**System architecture of InterMitoBase**.

The architecture of InterMitoBase is composed of three layers (Figure 3), the storage layer, the utility layer and the control layer. The storage layer which manages all the data in InterMitoBase is administrated by the relational database management system (RDBMS) of MySQL Community Server 5.2.3. The utility layer is implemented in the client-side through the web browser, in which HTML and Javascript/Ajax are used to generate the interactive user interface. The control layer is in the server-side where Perl scripts are used to handle processing logics, communicate with RDBMS, generate HTML source codes and trigger background programs. The web service is hosted on the HTTP server Apache 2.2.6. The network visualization is implemented by the tool of Graphviz 2.26 (http://www.graphviz.org/) and the statistical analysis is processed by R 2.12 (http://www.r-project.org/).

## Utility and Discussion

### Overview

**Figure 4:**
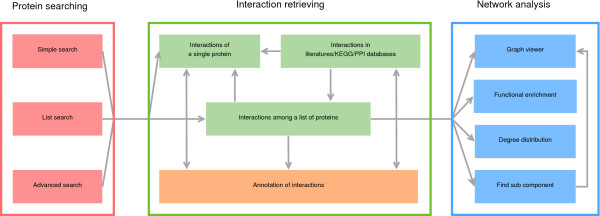
**Main contents of InterMitoBase**. InterMitoBase is composed of three sections, i.e., the protein searching section, the interaction retrieving section and the network analysis section.

InterMitoBase is designed for quick retrieving, visualizing and analyzing PPIs supporting human mitochondrial functions. It is composed of three sections, i.e., the searching section, the interaction retrieving section and the network analysis section (Figure 4).

### Searching Proteins of Interest

**Figure 5:**
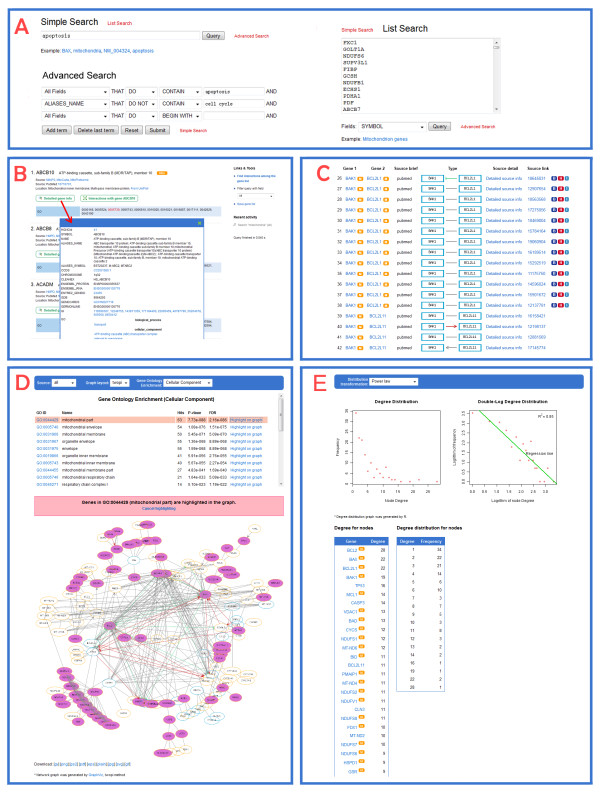
**Web pages in InterMitoBase**. A) Search page; B) Outcome page of the queried proteins; C) Record page of PPIs; D) Page for network visualization and functional analysis; E) Page for degree distribution.

InterMitoBase provides a search engine for users to source the proteins of interest. The queried keywords include various types of protein IDs, GO IDs and terms of biological functions/processes. 58 types of gene/protein identifiers used in the public databases such as GenBank, HGNC [14] and UniProt [15] are supported. Three types of searching, i.e., simple search, list search and advanced search, are supplied (Figure 5A). The searching system will return a page of the outcome proteins (Figure 5B), from which detailed information about the proteins, such as gene IDs, subcellular location, GO items and the related KEGG pathways, can be retrieved.

### Getting Protein-protein Interactions

The PPIs are navigated through the outcome page of proteins. Two retrieving processes are supported. One approach is to obtain all the interactions of a selected protein. The other approach is to get the interactions between all the outcome proteins. The retrieving returns a page where the outcome PPIs associated with related sources, directions and regulation types are also recorded (see Figure 5C).

### Network Visualization

The protein-protein network formed by the searched PPIs can be visualized graphically (see Figure 5D). The graph of over 300 proteins will not be illustrated since it is very time-consuming. However, its Graphviz file can be downloaded so that users can view the graph on a local machine.

### Functional Enrichment Analysis

InterMitoBase supports the functional enrichment analysis of proteins in a selected network (see Figure 5D). The analysis system returns the enriched GO terms, together with the p-values, the false discovery rates (FDR) and the numbers of related proteins in the network. Specifically, the proteins related to a selected GO term could be highlighted in the network graph.

### Degree Distribution

InterMitoBase also provides a general topological analysis on networks, i.e., the analysis of the degree distribution. Two transformations of the degree distribution (i.e., single log-plot and double log-plot) are given to judge whether the degree distribution is exponential or power-law (see Figure 5E). In addition, the degree and the degree frequency of each protein are listed.

## Conclusions

InterMitoBase is designed for quick retrieving, visualizing and analyzing PPIs that contribute to human mitochondrial functions. It integrates the most up-to-date PPIs for human mitochondria from diverse resources. Several on-line tools are also embedded to uncover the underlying biological properties of PPIs. Besides performing as an analysis platform and a PPI database, InterMitoBase will aid researchers aiming to obtain a comprehensive understanding of complex biological mechanisms underlying mitochondrial functions.

## Availability and Requirements

InterMitoBase is freely accessible at http://mcube.nju.edu.cn/bioinfo/intermitobase/ for academic or non-academic users. The client web browser should support JavaScript and Ajax to ensure full usage.

## Abbreviations

PPI: Protein-protein Interaction; KEGG: Kyoto Encyclopedia of Genes and Genomes; GO: Gene Ontology; FDR: False Discovery Rate.

## Authors' contributions

ZG and JL participated in the construction of the database and the draft of the manuscript. SG, MG and JW and XH carried out the manual curation on the data in InterMitoBase. JW and CZ conceived the study, design and coordination, and helped to draft the manuscript. All authors read and approved the final manuscript.
